# No Thesis, No Future! Exploring the associations between research inaccessibility and suicidal ideation

**DOI:** 10.1371/journal.pone.0345636

**Published:** 2026-03-20

**Authors:** Md. Abu Huraira, Md. Maruf Khan, Momotaj Begum, Pronab Das, Md. Omar Faruk, Sadikur Rahman, Sabrina Aktar, Md Zahidul Hasan, Moneerah Mohammad ALmerab, Remya Lathabhavan, Firoj Al-Mamun, Mohammed A. Mamun

**Affiliations:** 1 CHINTA Research Bangladesh, Dhaka, Bangladesh; 2 Department of Public Administration, Jahangirnagar University, Dhaka, Bangladesh; 3 Department of Biochemistry and Molecular Biology, Primeasia University, Dhaka, Bangladesh; 4 Department of Sociology, Noakhali Science and Technology University, Noakhali, Bangladesh; 5 One Health Institute, Chattogram Veterinary and Animal Sciences University, Chattogram, Bangladesh; 6 Department of Physiotherapy, Saic College of Medical Science & Technology, Dhaka, Bangladesh; 7 Department of Philosophy, Jahangirnagar University, Dhaka, Bangladesh; 8 Department of Library and Information Science, National University, Gazipur, Bangladesh; 9 Department of Chemical and Biomedical Engineering, University of Missouri, Columbia, Missouri, United States of America; 10 Department of Psychology, College of Education and Human Development, Princess Nourah Bint Abdulrahman University, Riyadh, Saudi Arabia; 11 Indian Institute of Management Bodh Gaya, Bodh Gaya, Bihar, India; 12 Department of Public Health, University of South Asia, Dhaka, Bangladesh; 13 School of Medicine, University of Nottingham, Nottingham, United Kingdom; School of Nursing Sao Joao de Deus, Evora University, PORTUGAL

## Abstract

**Background:**

Suicidal ideation is a pressing mental health concern among university students, especially in low- and middle-income countries like Bangladesh, where academic pressure, research-related stress, and limited support systems heighten the risk. While mental health conditions such as depression and anxiety are known predictors, the role of academic research involvement—particularly thesis engagement, supervision, and institutional support—remains underexplored and is examined in this study.

**Methods:**

A cross-sectional study was conducted among 508 university students and recent graduates in Bangladesh using a structured questionnaire. Data were analyzed using descriptive statistics, Chi-square tests, and binary logistic regression to examine associations between suicidal ideation and psychological, academic, and institutional factors, while QGIS was used to conduct geospatial analysis.

**Results:**

The prevalence of past-year suicidal ideation was 14.0%. In the adjusted model, anxiety (AOR = 2.41, 95% CI: 1.12–5.17) and insomnia (AOR = 3.29, 95% CI: 1.24–8.73) were significantly associated, while students not engaged in thesis work (AOR = 0.37, 95% CI: 0.16–0.88) and not interested in publishing their theses (AOR = 3.450, 95% CI: 1.047–11.371) had higher odds of suicidal ideation. Among students engaged in thesis work, unadjusted analyses revealed significant associations between suicidal ideation and intention to publish thesis work, poor supervisor availability, unclear academic guidance, lack of publication support, and perceptions of a non-supportive institutional research environment.

**Conclusion:**

Mental health challenges and academic exclusion—particularly non-thesis status and inadequate supervisory or institutional support—are key contributors to suicidal ideation. Universities should adopt inclusive research policies, enhance mental health services, and strengthen mentorship systems to reduce suicide risk and promote student well-being, especially in resource-constrained academic environments like Bangladesh.

## 1. Introduction

Suicide is a multifaceted phenomenon defined as the deliberate act of ending one’s own life, while suicidal behaviors encompass activities ranging from death wishes to attempted suicide [1]. Suicide ranks as the third leading cause of death among individuals aged 15–29 years across the world, with over 720,000 deaths annually and numerous additional suicide attempts [2]. The burden of suicide is disproportionately higher in low- and middle-income countries like Bangladesh (where the current study is conducted), accounting for nearly 80% of global cases [3]. Challenges such as academic pressures, intense competition, heavy workloads, and mental health issues (such as depression, anxiety, and suicidality in the extreme cases), increase vulnerability to suicidality among young adults, particularly university students, postgraduate researchers, and PhD candidates.

Globally, the prevalence of suicidal ideation among college students is estimated at 10.6% [4]. A meta-analysis among PhD students reported rates between 2–12% [5], while another study found 11.1% among medical students [6]. Bolotnyy et al. reported that suicidal ideation among PhD students is 3 times higher than in the general population (11.3% vs. 3.9%) [7]. Similarly, another study reported a 14.5% prevalence of past-year suicidal ideation among postgraduate researchers, with 4.3% at risk of attempting suicide and 37% at an elevated risk of suicide [8]. Furthermore, Hazell et al. found that over 40% of doctoral researchers experienced suicidal ideation within the past 12 months [9]. A survey by *The Guardian* reported self-harm tendencies in 11% of researchers and 16% of PhD students, emphasizing the mental health crisis within academic settings [10]. The pressures of academia disproportionately affect postgraduate researchers and PhD students, particularly during the later stages of their programs, with factors such as tight deadlines, publication demands, and supervisor relationships contributing significantly [7,8].

In Bangladesh, suicide ranks as the second leading cause of death after road accidents, with annual fatalities estimated between 10,000 and 14,000 individuals [11]. Although the overall number of student suicides declined from 585 in 2022–513 in 2023, suicides among university students increased from 85 to 98 during the same period, highlighting a growing mental health crisis within this vulnerable group [12,13]. Mamun et al. reported that 27 undergraduate students died by suicide between March 2020 and March 2021 during the COVID-19 pandemic, highlighting the inadequate mental health support systems in Bangladeshi universities [14]. While, several studies have reported past-year suicidal ideation prevalence rates of 13.4% [15], 14.7% [16], and 13.8% [17] among university students in Bangladesh. A systematic review found that the prevalence of suicidal ideation during the COVID-19 pandemic ranged from 5% to 19%, with trends suggesting a continued increase in the post-pandemic period [18]. Furthermore, a survey by the *Achol Foundation* involving 1,640 university students revealed that 34.15% had experienced suicidal ideation, reinforcing the urgent need for comprehensive and targeted mental health interventions at the tertiary education level [19].

University life often coincides with significant transitions, including increased responsibilities, financial stress, and academic pressures, which can exacerbate mental health challenges. Suicidal behaviors among university students are a complex issue influenced by various personal, social, and academic factors. In Bangladesh, a qualitative study reported that most public university students who committed suicide had a history of depression, family conflicts, relationship challenges, financial struggles, and academic stress [20], a finding supported by other studies [21]. Depression and anxiety have been consistently linked to suicidal ideation among Bangladeshi students [16], with one study reporting that students with depression were twice as likely to experience suicidal thoughts [17]. Another study found that 25.1% of students experienced suicidal ideation in the past month, with both depression and anxiety showing significant associations [22], a pattern also observed in studies conducted in the southern region of the country [23]. A meta-analysis reported a significant association between insomnia and suicide risk among university students [24], while Stevenson et al. found that students with insomnia were more prone to suicidal ideation compared to the general population [25].

These associations may be conceptually understood through the Stress–Diathesis Model of suicidal behavior, which posits that suicidal ideation emerges from the interaction between individual psychological vulnerabilities (e.g., anxiety, depression, and sleep disturbances) and environmental stressors [26,27]. According to this model, exposure to stress alone may not be sufficient to produce suicidal thoughts; rather, risk increases when stressors interact with pre-existing vulnerabilities. Prior research has consistently demonstrated associations between depression and anxiety and suicidal ideation among Bangladeshi university students [16,23], as well as between insomnia and suicide risk [24,25]. Within university settings, academic pressures, limited research access, and strained supervisory relationships may function as contextual stressors that interact with underlying vulnerabilities to increase suicide risk [8,28,29]. This model therefore provides a useful lens for examining how mental health conditions and academic research environments may jointly contribute to suicidal ideation among students.

In Bangladesh, research opportunities for university students are often limited, with research opportunities particularly restricted by academic performance and CGPA above 3.00 (out of 4.00) is mandatory to pursue a career in educational or banking sector [30]. Students who engage in thesis work or other research projects frequently encounter significant challenges, including inadequate funding, lack of mentorship, inadequate lab facilities, limited access to resources, and insufficient institutional support [31,32]. University professors in Bangladesh are more interested in administrative and political practices instead of conducting more impactful research work. Moreover, supervisors often imposed research topics to the research students and most of them were insulted by their supervisors during presenting their ideas and findings. Such behaviors from the professors often make the research students demotivated and hopeless to come up with new research challenges as well continuing in this field [33]. These problematic teacher-student dynamics are not unique to Bangladesh. Similar issues have been reported in other countries, where doctoral students often lack sufficient support and have no formal channels to report abusive supervision. As a consequent, they may feel powerless and compelled to comply with unreasonable demands from their supervisors [28]. These conditions frequently lead to delays in graduation and heightened psychological distress. Previous studies have established a direct link between abusive supervision and suicidal ideation, particularly among graduate students in China [29].

While several studies have explored the predictors of suicidal ideation among university students in Bangladesh, few have specifically investigated how academic research experiences—such as thesis involvement, supervisor support, or institutional research resources—relate to mental health outcomes. Most prior studies have focused on general academic stress or sociodemographic factors, rather than the distinct pressures associated with research-oriented academic tracks. This study aims to address this gap by assessing the prevalence of suicidal ideation and identifying academic and research-related risk factors among university students and recent graduates. By integrating data on research environment variables, supervisor interactions, and thesis status, the findings are expected to provide targeted insights into the academic drivers of suicidality. These results may inform the development of more supportive and equitable research environments for student researchers in Bangladesh.

## 2. Methods

### 2.1 Study design and participants

This study was focused on assessing the prevalence and risk factors of suicidal ideation among those who engaged in academic research. Only the current students or recent graduates from various universities in Bangladesh were considered as eligible for this study. To ensure participants had some forms of academic research exposure, participants in at least 3^rd^ year of their undergraduate studies were considered in this study. A comprehensive literature review was utilized for questionnaire development and then pretested with 20 students between February 19 and March 6, 2024. The final online survey consists of four sections including socio-demographic, academic, research-related, and mental health conditions (depression, anxiety, and insomnia), and suicidal ideation.

### 2.2 Data collection procedure

Data were collected using a non-probability sampling technique (combining convenience and snowball sampling) via an online questionnaire distributed through *Google Forms*. Data collection occurred from March 6–12, 2024, through *Google Forms*, disseminated via multiple online platforms, including emails, messages, and posts on the principal investigator’s institute’s Facebook page, as well as educational forums. In addition, the survey link was shared through the social media profiles of the participants and the researchers involved in the project. To further encourage participation, a free webinar titled ‘Research and Higher Study’ was organized by principal investigator’s institute, and attendees were invited to participate in the study. Informed consent was obtained at the beginning of the questionnaire, with participants given detailed information about the survey’s objectives and ethical considerations. Finally, a total of 522 responses were initially received, with 508 responses being included in the final analysis after data cleaning to meet eligibility criteria (e.g., ensuring the respondent was a Bangladeshi student or graduate and excluding incomplete responses).

### 2.3 Measures

#### 2.3.1 Sociodemographic factors.

A variety of sociodemographic factors such as gender, age, marital status, permanent and current living place, financial support sources, socioeconomic status, personal income level, family research support, and presence of a researcher within the family were collected in this survey. Four distinct groups were formed Based on their income level: lower class (< 20,000 BDT), middle class (21,000–40,000 BDT), high class (41,000–60,000 BDT), and upper high class (> 61,000 BDT). Respondents’ relationships with the researchers (if there is any within the family) were also classified where relatives such as uncles, cousins, brothers-in-law, sisters-in-law, and fathers-in-law were included in ‘others’ category.

#### 2.3.2 Academic information.

A series of questions concerning the respondents educational background were also asked in this study. These questions typically included their university name, field of study for bachelor’s and master’s degree, whether they continued both degrees at the same university or not, presence of any ‘session jams’ (refers to the academic delays that prevent degrees from being completed within due time), and their GPA or CGPA for both degrees. Universities were divided into three distinct groups: specialized universities (e.g., universities of science and technology, engineering, and agriculture), health sciences (e.g., nursing and medical institutions), and others. Furtherly, science (e.g., agriculture, environmental science, engineering), life sciences (e.g., food and nutrition), and health sciences (e.g., public health, nursing, and medicine) were the three categories to report respondents’ field of study.

#### 2.3.3 Research related variables.

Respondents were questioned whether they had research course(s) during their bachelor program and/or outside university education. Their satisfaction with these research courses were assessed by utilizing a Likert scale with four-points (ranging from ‘not at all’ to ‘highly satisfied’). Participants were further divided into two groups: thesis group (those who had either completed or were in the midst of completing a thesis) and non-thesis group (those who had not taken a thesis owing to indecision, lack of opportunity, or unwillingness, etc.). Participants were asked whether they had any research project engagement except their thesis. Additionally, their involvement with research-related professions and their desire to pursue research related careers were also recorded.

#### 2.3.4 Research environment.

To assess the influence of the academic research environment on suicidal ideation, additional items were administered to participants who had completed or were currently engaged in thesis work (n = 268).

First, thesis-related variables included thesis type, source of funding, the primary intention behind undertaking a thesis (i.e., degree requirement *vs.* research career), and interest in publishing the thesis work. Second, the quality of thesis supervision and institutional research support was assessed using three-point Likert scales adapted to each item (e.g., from ‘not at all’ to ‘to a great extent’, or ‘not satisfactory’ to ‘excellent’). A total of 10 supervisor-related variables were included, covering availability, responsiveness, frequency of interaction, clarity of guidance, and support across various research stages (e.g., literature review, methodology development, data analysis, proposal/ thesis drafting, and publication preparation). Additionally, five institutional support variables were used to assess perceptions of supportive environment within the universities or institutes, students’ overall satisfaction with the supports from the faculty members, peer and senior researcher connections, availability and access to the resources, and opportunity to participate in relevant training or workshops.

#### 2.3.5 Mental health problems.

The Patient Health Questionnaire (PHQ-4) was used to evaluate depression and anxiety levels among participants [34]. PHQ-4 consists of two subscales: PHQ-2 for depression and GAD-2 for anxiety. Each item was rated on a 4-point Likert scale (0 = not at all, 1 = several days, 2 = more than half the days, 3 = nearly every day) over = previous week. Cut-off scores ≥ 3 were used to indicate significant depression or anxiety symptoms, with a sensitivity and specificity of 82.9% and 90%, and 86% and 83%, respectively. The internal consistency (Cronbach’s alpha) for this scale was 0.70. While, insomnia was assessed using two items from the Insomnia Severity Index (ISI-2), rated on a 5-point Likert scale (0 = very satisfied to 4 = very dissatisfied). A cut-off score of ≥ 6 indicated significant insomnia symptoms, with a sensitivity of 84% and a specificity of 76% [35]. The Cronbach’s alpha for this scale was 0.75. To assess suicidal ideation, questions used in previous studies were utilized (i.e., binary ‘yes/no’ responses).

#### 2.3.6 Suicidal ideation.

Suicidal ideation was assessed using a single-item, self-report question designed to identify serious contemplation of suicide within the past year. Participants were asked: *“In the past year, when you were extremely emotionally or mentally distressed—due to personal, academic, family, or other life circumstances—have you ever seriously thought about ending your life?”* Responses were recorded in a binary format (“Yes” or “No”), with “Yes” responses coded as indicative of suicidal ideation. This single-item screening approach has been widely employed in epidemiological and cohort studies to assess suicidal ideation, particularly among adolescents and young adults [36–38]. It allows for efficient identification of individuals experiencing significant psychological distress while minimizing participant burden. However, while single-item measures are practical for large-scale studies, they may not capture the full spectrum or intensity of suicidal thoughts, such as frequency, duration, or presence of a suicide plan.

### 2.4 Ethical considerations

Prior to the study’s commencement, ethical approval was obtained from the Institutional Review Board at the Patuakhali Science and Technology University, Bangladesh [Reference Number: PSTU/IEC/2023/81]. The study complied with the ethical guidelines set forth in the revised Helsinki Declaration of 2013, ensuring the protection of human participants. Participants were provided with a clear explanation of the study’s objectives at the start of the questionnaire, and informed written consent was obtained. They were also informed of their right to withdraw from the study at any time without any repercussions. No financial or non-financial incentives were offered for participation.

### 2.5 Statistical analysis

Data were collected via Google Forms and initially exported to Microsoft Excel for data cleaning and formatting. The cleaned dataset was then imported into SPSS version 25 for statistical analyses.

Descriptive statistics were computed to summarize the characteristics of the study sample. Frequencies and percentages were calculated for categorical variables, while appropriate measures of central tendency were used for continuous variables. These analyses covered sociodemographic characteristics, academic status, thesis-related factors, mental health variables, and research environment characteristics.

Inferential statistics were used to examine associations between study variables and suicidal ideation. Chi-square tests were applied to assess bivariate associations among categorical variables. Subsequently, binary logistic regression analysis was conducted to identify factors independently associated with suicidal ideation, which was treated as the dependent variable (coded as 1 = Yes, 0 = No). Variables included in the multivariable models were selected based on theoretical relevance and prior empirical literature, rather than solely on bivariate statistical significance. Adjusted odds ratios (AORs) with 95% confidence intervals (CIs) were reported, and a two-tailed p-value < 0.05 was considered statistically significant.

Variables related to thesis characteristics, supervisor support, and the institutional research environment were applicable only to participants who had completed or were currently engaged in thesis work (n = 268). Accordingly, statistical analyses involving these variables, such as chi-square tests and binary logistic regression, were conducted exclusively within this subgroup.

Geospatial analysis was conducted to visualize the regional distribution of suicidal ideation across Bangladesh’s eight administrative divisions using QGIS Desktop version 3.32.3. Administrative boundary shapefiles for Bangladesh were obtained from an openly licensed source and used solely for visualization purposes. This analysis was performed for descriptive and exploratory purposes only, and no inferential or causal conclusions were drawn from the observed spatial patterns.

## 3. Results

### 3.1 Characteristics of the study participants

Among the 508 participants, 55.7% were male, with a mean age of 25.61 years (± 3.124). The majority were aged between 24 and 26 years. Most participants were unmarried (76.4%), and 61.4% came from rural areas. About 39.2% were living with their family members, and for 44.4%, the family was the main source of financial support. Additionally, 58.5% were self-employed. A significant proportion (37.1%) had completed their bachelor’s degrees from general public or private institutions, and the largest academic group (35.1%) belonged to Social Science and Arts disciplines.

Regarding mental health conditions, the prevalence of depression, anxiety, and insomnia were 39.8%, 29.3%, and 12.2%, respectively (Table 1). Additionally, 14.0% of the participants reported experiencing suicidal ideation in the past year.

**Table 1:** Description of study variables and their associations with suicidal ideation among university students (N = 508).

Variables	Total (n; %)	Suicidal Ideation		Chi-square Test Statistics		
		Yes	No	χ^2^ test value	df	*p*-value
**Socio-demographic Factors**						
**Gender**						
Male	283; 55.7%	34; 12.0%	249; 88.0%	2.046	1	0.153
Female	225; 44.3%	37; 16.4%	188; 83.6%			
**Age Group**						
19–23 years	97; 19.1%	17; 17.5%	80; 82.5%	1.621	2	0.445
24–26 years	274; 53.9%	38; 13.9%	236; 86.1%			
27 and above	137; 27.0%	16; 11.7%	121; 88.3%			
**Marital Status**						
Married	120; 23.6%	15; 12.5%	105; 87.5%	0.285	1	0.594
Unmarried	388; 76.4%	56; 14.4%	332; 85.6%			
**Place of Permanent Residence**						
Rural	312; 61.4%	39; 12.5%	273; 87.5%	1.466	1	0.226
Urban	196; 38.6%	32; 16.3%	164; 83.7%			
**Currently Living with Family**						
Yes	199; 39.2%	28; 14.1%	171; 85.9%	0.002	1	0.961
No	309; 60.8%	43; 13.9%	266; 86.1%			
**Source of Financial Support**						
Own	176; 35.1%	23; 13.1%	153; 86.9%	0.261	2	0.878
Family	223; 44.4%	33; 14.8%	190; 85.2%			
Both	103; 20.5%	15; 14.6%	88; 85.4%			
**Socio-Economic Status**						
Lower class	98; 20%	12; 12.2%	86; 87.8%	1.324	2	0.516
Middle class	147; 29.9%	18; 12.2%	129; 87.8%			
High class	246; 50.1%	39; 15.9%	207; 84.1%			
**Self-Employment Status**						
Yes	297; 58.5%	44; 14.8%	253; 85.2%	0.418	1	0.518
No	211; 41.5%	27; 12.8%	184; 87.2%			
**Type of Institution Attended for Bachelor’s Degree**						
General	187; 37.1%	34; 18.2%	153; 81.8%	7.317	3	0.062
Private	62; 12.3%	10; 16.1%	52; 83.9%			
Specialized	153; 30.4%	13; 8.5%	140; 91.5%			
Health Sciences	102; 20.2%	12; 11.8%	90; 88.2%			
**Academic Discipline of Bachelor’s Degree**						
Life Sciences	156; 30.8%	23; 14.7%	133; 85.3%	1.085	3	0.781
Health Sciences	134; 26.4%	16; 11.9%	118; 88.1%			
Social Sciences and Arts	178; 35.1%	24; 13.5%	154; 86.5%			
Business and others	39; 7.7%	7; 17.9%	32; 82.1%			
**Research Exposure and Family Support**						
**Family Support for Academic Research**						
Yes	405; 79.7%	52; 12.8%	353; 87.2%	2.147	1	0.143
No	103; 20.3%	19; 18.4%	84; 81.6%			
**Presence of Researcher in Family**						
Yes	82; 16.1%	10; 12.2%	72; 87.8%	0.258	1	0.611
No	426; 83.9%	61; 14.3%	365; 85.7%			
**Attended Research Course During Bachelor’s Program**						
Yes	372; 73.2%	46; 12.4%	326; 87.6%	2.999	1	0.083
No	136; 26.8%	25; 18.4%	111; 81.6%			
**Satisfaction with University Research Courses**						
Yes	220; 52.0%	27; 12.3%	193; 87.7%	1.714	1	0.190
No	203; 48.0%	34; 16.7%	169; 83.3%			
**Completion of External Research Course**						
Yes	99; 19.5%	17; 17.2%	82; 82.8%	1.044	1	0.307
No	409; 80.5%	54; 13.2%	355; 86.8%			
**Engagement in Thesis Work**						
Thesis group	268; 52.8%	32; 11.9%	236; 88.1%	1.956	1	0.162
Non-thesis group	240; 47.2%	39; 16.3%	201; 83.8%			
**Participation in Other Research Projects (Beyond Thesis)**						
Yes	128; 25.2%	22; 17.2%	106; 82.8%	1.441	1	0.230
No	379; 74.8%	49; 12.9%	330; 87.1%			
**Current Involvement in Research-Oriented Job or Role**						
Yes	186; 44.7%	21; 11.3%	165; 88.7%	1.361	1	0.243
No	230; 55.3%	35; 15.2%	195; 84.8%			
**Aspiration to Pursue Research as a Career**						
Yes	307; 60.4%	42; 13.7%	265; 86.3%	0.056	1	0.812
No	201; 39.6%	29; 14.4%	172; 85.6%			
**Mental Health Problems**						
**Symptoms of Depression**						
Yes	202; 39.8%	42; 20.8%	160; 79.2%	12.957	1	**<0.001**
No	306; 60.2%	29; 9.5%	277;90.5%			
**Symptoms of Anxiety**						
Yes	149; 29.3%	36; 24.2%	113; 75.8%	18.190	1	**<0.001**
No	359; 70.7%	35; 9.7%	324; 90.3%			
**Symptoms of Insomnia**						
Yes	62; 12.2%	19; 30.6%	43; 69.4%	16.320	1	**<0.001**
No	446; 87.8%	52; 11.7%	394; 88.3%			

### 3.2 Description of research exposure and family support

Approximately 83.9% of the respondents reported that they were the only person in their family engaged in research activities. However, 79.7% stated that they received support from their family for their research endeavors. About 73.2% had taken at least one research course during their undergraduate studies. Among them, 52.0% were satisfied with the course(s), while 48.0% expressed dissatisfaction. Additionally, 19.5% of the respondents had completed at least one research course outside of their university.

The majority of participants (52.8%) had either completed or were in the process of completing a thesis. Nearly one-fourth (25.2%) reported involvement in research beyond their thesis. Furthermore, 44.7% were engaged in research-related professions, and 60.4% expressed interest in pursuing a research-related career (Table 1).

### 3.3 Description of thesis and research environment-related variables

Out of the total sample, 268 participants had completed or were currently engaged in thesis work. Among them, a majority (80.6%) reported receiving no financial support for their thesis. More than half (55.2%) undertook the thesis as a mandatory degree requirement, and 23.1% showed no interest in publishing their work.

Regarding supervisor support, 79.8% rated their supervisor’s availability as satisfactory or better, and 89.1% found the guidance they received to be clear. Regular or occasional meetings with supervisors were reported by 84.0%, and over 84% received support with literature reviews, methodology development, and data analysis. However, only 49.6% reported receiving regular feedback on their thesis drafts. In terms of publication support, 81.3% were satisfied with their supervisor’s assistance, 76.1% received help with journal selection, and 77.7% acknowledged their supervisor’s role in facilitating thesis publication.

Regarding institutional support, 85.4% perceived a somewhat supportive research environment. Faculty support was rated satisfactory by 80.2%, 73.9% benefited from peer and senior researcher networks, 66.8% had adequate access to research resources, and 50.7% had participated in relevant training or workshops (Table 2).

**Table 2:** Thesis and research environment-related variables and their associations with suicidal ideation (n = 268).

Variables	Total (n; %)	Suicidal Ideation		Chi-square Test Statistics		
		Yes	No	χ^2^ test value	df	*p*-value
**Thesis Related Variables**						
**Received Funding for Thesis Research**						
No	216; 80.6%	27; 12.5%	189; 87.5%	0.332	1	0.565
Yes	52; 19.4%	5; 9.6%	47; 90.4%			
**Motivation for Undertaking Thesis Work**						
For degree	148; 55.2%	14; 9.5%	134; 90.5%	1.935	1	0.164
For research career	120; 44.8%	18; 15.0%	102; 85.0%			
**Nature of the Thesis Project**						
Cross-sectional study	76; 28.4%	10; 13.2%	66; 86.8%	0.920	3	0.821
Experimental study	76; 28.4%	7; 9.2%	69; 90.8%			
Case-report	72; 26.9%	10; 13.9%	62; 86.1%			
Others	44; 16.4%	5; 11.4%	39; 88.6%			
**Intention to Publish Thesis Work**						
Not interested	62; 23.1%	12; 19.4%	50; 80.6%	4.217	1	**0.040**
Interested	206; 76.9%	20; 9.7%	186; 90.3%			
**Supervisor’s Support Related Variables**						
**Supervisor’s Availability and Responsiveness**						
Not satisfactory	54; 20.1%	14; 25.9%	40; 74.1%	13.857	2	**0.001**
Satisfactory	115; 42.9%	7; 6.1%	108; 93.9%			
Very satisfactory	99; 36.9%	11; 11.1%	88; 88.9%			
**Frequency of Meetings with Supervisor**						
Rarely	43; 16.0%	6; 14.0%	37; 86.0%	1.124	2	0.570
Occasionally	101; 37.7%	14; 13.9%	87; 86.1%			
Regularly	124; 46.3%	12; 9.7%	112; 90.3%			
**Clarity and Helpfulness of Supervisor’s Guidance**						
Not clear at all	29; 10.8%	8; 27.6%	21; 72.4%	7.998	2	**0.018**
Somewhat clear	84; 31.3%	10; 11.9%	74; 88.1%			
Clear	155; 57.8%	14; 9.0%	141; 91.0%			
**Supervisor Support for Literature Review and Background Research**						
Not at all	41; 15.3%	9; 22.0%	32; 78.0%	4.780	2	0.092
Small to moderate	176; 65.7%	17; 9.7%	159; 90.3%			
To a great extent	51; 19.0%	6; 11.8%	45; 88.2%			
**Supervisor Assistance in Methodology and Analysis Plan Development**						
Not at all	40; 14.9%	9; 22.5%	31; 77.5%	5.081	2	0.079
Small to moderate	172; 64.2%	18; 10.5%	154; 89.5%			
To a great extent	56; 20.9%	5; 8.9%	51; 91.1%			
**Supervisor Support in Refining Methods and Conducting Data Analysis**						
Not at all	35; 13.1%	8; 22.9%	27; 77.1%	4.642	2	0.098
Small to moderate	171; 63.8%	17; 9.9%	154; 90.1%			
To a great extent	62; 23.1%	7; 11.3%	55; 88.7%			
**Frequency of Feedback on Proposals, Papers, and Thesis Drafts**						
Rarely	41; 15.3%	8; 19.5%	33; 80.5%	3.373	2	0.185
Occasionally	94; 35.1%	12; 12.8%	82; 87.2%			
Regularly	133; 49.6%	12; 9.0%	121; 91.0%			
**Satisfaction with Supervisor Feedback on Publication Readiness**						
Not satisfactory	50; 18.7%	8; 16.0%	42; 84.0%	2.606	2	0.272
Satisfactory	82; 30.6%	12; 14.6%	70; 85.4%			
Very satisfactory	136; 50.7%	12; 8.8%	124; 91.2%			
**Satisfaction with Supervisor Guidance on Conference/Journal Selection**						
Not satisfactory	64; 23.9%	11; 17.2%	53; 82.8%	2.202	2	0.333
Satisfactory	68; 25.4%	7; 10.3%	61; 89.7%			
Very satisfactory	136; 50.7%	14; 10.3%	122; 89.7%			
**Supervisor’s Influence on Research Publication Outcomes**						
Not influence	60; 22.4%	11; 18.3%	49; 81.7%	6.260	2	**0.044**
Influence	147; 54.9%	11; 7.5%	136; 92.5%			
High influence	61; 22.8%	10; 16.4%	51; 83.6%			
**Institutional Support Related Variables**						
**Perception of a Supportive Institutional Research Environment**						
Not at all	39; 14.6%	7; 17.9%	32; 82.1%	6.615	2	**0.037**
Small to moderate	186; 69.4%	16; 8.6%	170; 91.4%			
To a great extent	43; 16.0%	9; 20.9%	34; 79.1%			
**Satisfaction with Faculty Support for Research Activities**						
Not satisfactory	53; 19.8%	11; 20.8%	42; 79.2%	5.221	2	0.074
Satisfactory	96; 35.8%	8; 8.3%	88; 91.7%			
Very satisfactory	119; 44.4%	13; 10.9%	106; 89.1%			
**Satisfaction with Peer and Senior Researcher Networking Opportunities**						
Not satisfactory	70; 26.1%	13; 18.6%	57; 81.4%	5.947	2	0.051
Satisfactory	96; 35.8%	6; 6.3%	90; 93.8%			
Very satisfactory	102; 38.1%	13; 12.7%	89; 87.3%			
**Satisfaction with Access to Institutional Research Resources**						
Not satisfactory	89; 33.2%	12; 13.5%	77; 86.5%	1.771	2	0.413
Satisfactory	77; 28.7%	6; 7.8%	71; 92.2%			
Very satisfactory	102; 38.1%	14; 13.7%	88; 86.3%			
**Access to Research-Related Institutional Training and Workshops**						
Not at all	132; 49.3%	20; 15.2%	112; 84.8%	2.831	2	0.243
Somewhat	68; 25.4%	7; 10.3%	61; 89.7%			
Enough	68; 25.4%	5; 7.4%	63; 92.6%			

### 3.4 Associations between study variables and suicidal ideation

Chi-square analyses showed that no socio-demographic variables were significantly associated with past-year suicidal ideation. However, several mental health and academic factors showed significant associations. Among participants with depression, 20.8% reported suicidal ideation, compared to 9.5% of those without depression (χ² = 12.957, p < 0.001). Suicidal ideation was also higher among those with anxiety symptoms (24.2% vs. 9.7%; χ² = 18.190, p < 0.001) and insomnia (30.6% vs. 12.1%; χ² = 14.11, p < 0.001). While, suicidal ideation was more common among those who had not completed or were not currently engaged in a thesis (16.3%) than among those who were (11.9%; χ² = 1.956, p = 0.162) engaged in a thesis project. Participants not interested in a research-related career also had a higher prevalence of suicidal ideation (14.4%) compared to those with such interest (13.7%; χ² = 0.056, *p* = 0.812) (Table 1).

Among participants who had completed or were currently engaged in thesis work, several aspects of the research environment and supervisory support were significantly associated with past-year suicidal ideation. Students who intended to publish their thesis had a higher prevalence of suicidal ideation (19.4%) compared to those not interested in publishing (9.7%; χ² = 4.217, p = 0.040). Those who rated their supervisor’s availability as unsatisfactory reported markedly higher suicidal ideation (25.9%) than those who found it satisfactory (6.1%; χ² = 13.857, p = 0.001). Similarly, participants who described their supervisor’s guidance as unclear reported the highest rate of suicidal ideation (27.6%) compared to those who found the guidance clear (9.0%; χ² = 7.998, p = 0.018). Students who perceived no supervisor influence on thesis publication also reported more suicidal ideation (18.3%) than those who did (7.5%; χ² = 6.260, p = 0.044). Regarding institutional factors, students who perceived no supportive research environment had higher suicidal ideation (17.9%) than those reporting moderate (8.6%) or strong support (20.9%; χ² = 6.615, p = 0.037). While other supervisory and institutional factors were not statistically significant, several showed directional trends that may warrant further investigation (Table 3).

**Table 3:** Binary logistic regression analysis of factors associated with past-year suicidal ideation (N = 508).

Variables	Unadjusted Model			Adjusted Model		
	Odds Ratio (OR)	95% Confidence Interval (CI)	*p*-value	Adjusted Odds Ratio (AOR)	95% Confidence Interval (CI)	*p*-value
**Socio-demographic Factors**						
**Gender**						
Male	0.694	0.420-1.147	0.154	0.488	0.222-1.075	0.075
Female	Reference			Reference		
**Age Group**						
19–23 years	1.607	0.768-3.364	0.448	4.426	1.062-18.436	0.120
24–26 years	1.218	0.653-2.272		2.058	0.672-6.303	
27 and above	Reference			Reference		
**Marital Status**						
Married	0.847	0.460-1.560	0.594	1.512	0.569-4.021	0.407
Unmarried	Reference			Reference		
**Place of Permanent Residence**						
Rural	0.732	0.441-1.214	0.227	0.645	0.297-1.401	0.268
Urban	Reference			Reference		
**Currently Living with Family**						
Yes	1.013	0.606-1.692	0.961	0.962	0.404-2.292	0.930
No	Reference			Reference		
**Source of Financial Support**						
Own	0.882	0.437-1.778	0.878	1.353	0.451-4.061	0.594
Family	1.019	0.526-1.973		0.655	0.160-2.679	
Both	Reference			Reference		
**Socio-Economic Status**						
Lower class	0.741	0.370-1.483	0.518	0.648	0.231-1.818	0.593
Middle class	0.741	0.406-1.350		0.667	0.265-1.678	
High class	Reference			Reference		
**Self-Employment Status**						
Yes	1.185	0.708-1.984	0.518	0.877	0.252-3.053	0.837
No	Reference			Reference		
**Research Exposure and Family Support**						
**Family Support for Academic Research**						
Yes	0.651	0.366-1.159	0.145	0.559	0.231-1.355	0.198
No	Reference			Reference		
**Presence of Researcher in Family**						
Yes	0.831	0.407-1.699	0.612	0.538	0.193-1.497	0.235
No	Reference			Reference		
**Type of Institution Attended for Bachelor’s Degree**						
General	1.667	0.821-3.382	0.070	0.367	0.046-2.943	0.253
Private	1.442	0.583-3.569		0.631	0.085-4.697	
Specialized	0.696	0.304-1.594		0.168	0.017-1.694	
Health Sciences	Reference			Reference		
**Academic Discipline of Bachelor’s Degree**						
Life Sciences	0.791	0.312-2.003	0.783	1.307	0.329-5.197	0.560
Health Sciences	0.620	0.235-1.636		0.341	0.042-2.763	
Social Sciences and Arts	0.712	0.283-1.795		1.120	0.271-4.619	
Business and others	Reference			Reference		
**Attended Research Course During Bachelor’s Program**						
Yes	0.627	0.368-1.067	0.085	0.691	0.284-1.683	0.416
No	Reference			Reference		
**Satisfaction with University Research Courses**						
Yes	0.695	0.403-1.200	0.192	0.772	0.359-1.658	0.507
No	Reference			Reference		
**Completion of External Research Course**						
Yes	1.363	0.751-2.473	0.308	1.729	0.676-4.419	0.253
No	Reference			Reference		
**Engagement in Thesis Work**						
Thesis group	0.699	0.422-1.157	0.163	0.374	0.158-0.882	**0.025**
Non-thesis group	Reference			Reference		
**Participation in Other Research Projects (Beyond Thesis)**						
Yes	1.398	0.808-2.419	0.231	2.041	0.842-4.945	0.114
No	Reference			Reference		
**Current Involvement in Research-Oriented Job or Role**						
Yes	0.709	0.397-1.266	0.245	0.867	0.335-2.245	0.769
No	Reference			Reference		
**Aspiration to Pursue Research as a Career**						
Yes	0.940	0.564-1.566	0.812	0.793	0.341-1.842	0.589
No	Reference			Reference		
**Mental Health Problems**						
**Symptoms of Depression**						
Yes	2.507	1.503-4.182	**<0.001**	1.335	0.612-2.915	0.468
No	Reference			Reference		
**Symptoms of Anxiety**						
Yes	2.949	1.767-4.922	**<0.001**	2.408	1.122-5.167	**0.024**
No	Reference			Reference		
**Symptoms of Insomnia**						
Yes	3.348	1.815-6.177	**<0.001**	3.290	1.241-8.728	**0.017**
No	Reference			Reference		

### 3.5 Factors associated with suicidal ideation in the adjusted logistic regression model

In the adjusted logistic regression model, three variables were significantly associated with past-year suicidal ideation. Participants who reported experiencing anxiety had more than twice the odds of suicidal ideation compared to those without anxiety (AOR = 2.41, 95% CI: 1.122–5.167, *p* = 0.024), while those with insomnia had over three times higher odds (AOR = 3.29, 95% CI: 1.241–8.728, *p* = 0.017). Additionally, participants engaged in thesis work had significantly lower odds of suicidal ideation than their non-thesis counterparts (AOR = 0.374, 95% CI: 0.158–0.882, *p* = 0.025). Other variables were not significantly associated with suicidal ideation in the adjusted model; however, gender approached statistical significance (AOR = 0.488, 95% CI: 0.222–1.075, *p* = 0.075) but did not meet the conventional threshold. The model demonstrated good fit based on the Hosmer and Lemeshow test (χ² = 8.587, df = 8, *p* = 0.378) (Table 3).

In the adjusted logistic regression model, only one variable from thesis related variables remained significantly associated with past-year suicidal ideation: students who were not interested in publishing their thesis had significantly higher odds of suicidal ideation compared to those who were interested (AOR = 3.450, 95% CI: 1.047–11.371, *p* = 0.042). While students reporting unsatisfactory supervisor availability had increased odds of suicidal ideation (AOR = 7.853, 95% CI: 0.962–64.105, *p* = 0.013), the wide confidence interval indicates uncertainty and should be interpreted cautiously. Similarly, those with limited participation in research training or workshops showed higher odds (AOR = 4.499, 95% CI: 1.074–18.845, *p* = 0.116), though the association was not statistically significant. Other variables related to supervision quality and institutional resource support did not show statistically significant associations after adjustment. The model demonstrated good fit based on the Hosmer and Lemeshow test (χ² = 5.292, df = 8, *p* = 0.726) (Table 4).

**Table 4:** Binary logistic regression analysis of thesis and research environment-related factors of suicidal ideation among thesis students (n = 268).

Variables	Unadjusted Model			Adjusted Model		
	Odds Ratio (OR)	95% Confidence Interval (CI)	*p*-value	Adjusted Odds Ratio (AOR)	95% Confidence Interval (CI)	*p*-value
**Thesis Related Variables**						
**Received Funding for Thesis Research**						
No	0.745	0.272-2.037	0.566	0.568	0.131-2.475	0.452
Yes	Reference			Reference		
**Motivation for Undertaking Thesis Work**						
For degree	0.592	0.281-1.246	0.168	0.687	0.228-2.069	0.504
For research career	Reference			Reference		
**Nature of the Thesis Project**						
Cross-sectional study	1.182	0.376-3.711	0.823	1.046	.226-4.854	0.788
Experimental study	0.791	0.235-2.661		.652	.116-3.666	
Case-report	1.258	0.400-3.957		1.352	.308-5.938	
Others	Reference			Reference		
**Intention to Publish Thesis Work**						
Not interested	2.232	1.022-4.873	**0.044**	3.450	1.047 −11.371	**0.042**
Interested	Reference			Reference		
**Supervisor Support Related Variables**						
**Supervisor’s Availability and Responsiveness**						
Not satisfactory	2.800	1.169-6.708	**0.002**	7.853	.962-64.105	**0.013**
Satisfactory	0.519	0.193-1.393		0.616	.130-2.915	
Very satisfactory	Reference			Reference		
**Frequency of Meetings with Supervisor**						
Rarely	1.514	0.531-4.317	0.573	0.320	.0342-2.996	0.069
Occasionally	1.502	0.661-3.411		2.043	.398-10.487	
Regularly	Reference			Reference		
**Clarity and Helpfulness of Supervisor’s Guidance**						
Not clear at all	3.837	1.437-10.245	**0.026**	3.573	.258-49.532	0.595
Somewhat clear	1.361	0.577-3.213		1.356	.232-7.925	
Clear	Reference			Reference		
**Supervisor Support for Literature Review and Background Research**						
Not at all	2.109	0.683-6.517	0.103	1.157	0.080-16.635	0.930
Small to moderate	0.802	0.299-2.153		0.852	0.102-7.129	
To a great extent	Reference			Reference		
**Supervisor Assistance in Methodology and Analysis Plan Development**						
Not at all	2.961	0.909-9.645	0.091	4.144	0.262-65.472	0.405
Small to moderate	1.192	0.421-3.374		4.108	0.522-32.362	
To a great extent	Reference			Reference		
**Supervisor Support in Refining Methods and Conducting Data Analysis**						
Not at all	2.328	0.764-7.093	0.112	0.246	0.013-4.723	0.467
Small to moderate	0.867	0.341-2.204		0.264	0.032-2.197	
To a great extent	Reference			Reference		
**Frequency of Feedback on Proposals, Papers, and Thesis Drafts**						
Rarely	2.444	0.923-6.473	0.196	1.555	0.171-14.113	0.740
Occasionally	1.476	0.632-3.445		2.016	0.336-12.111	
Regularly	Reference			Reference		
**Satisfaction with Supervisor Feedback on Publication Readiness**						
Not satisfactory	1.968	0.753-5.144	0.280	0.461	0.028-7.580	0.471
Satisfactory	1.771	0.756-4.153		1.544	0.225-10.579	
Very satisfactory	Reference			Reference		
**Satisfaction with Supervisor Guidance on Conference/Journal Selection**						
Not satisfactory	1.809	0.771-4.244	0.340	0.827	0.073-9.380	0.985
Satisfactory	1.000	0.384-2.606		0.990	0.168-5.823	
Very satisfactory	Reference			Reference		
**Supervisor’s Influence on Research Publication Outcomes**						
Not influence	1.145	0.446-2.936	0.051	0.331	0.042-2.625	0.084
Influence	0.413	0.165-1.030		0.179	0.036 −0.886	
High influence	Reference			Reference		
**Institutional Support Related Variables**						
**Perception of a Supportive Institutional Research Environment**						
Not at all	0.826	0.275-2.481	**0.043**	0.243	0.029-2.057	0.258
Small to moderate	0.356	0.145-0.871		0.317	0.078-1.298	
To a great extent	Reference			Reference		
**Satisfaction with Faculty Support for Research Activities**						
Not satisfactory	2.136	0.887-5.143	0.083	1.298	0.144-11.371	0.973
Satisfactory	0.741	0.294-1.869		1.177	0.194-7.132	
Very satisfactory	Reference			Reference		
**Satisfaction with Peer and Senior Researcher Networking Opportunities**						
Not satisfactory	1.561	0.676-3.608	0.062	1.268	0.179-9.001	0.263
Satisfactory	0.456	0.166-1.254		0.388	0.074-2.050	
Very satisfactory	Reference			Reference		
**Satisfaction with Access to Institutional Research Resources**						
Not satisfactory	0.980	0.427-2.245	0.422	0.205	0.033-1.274	0.176
Satisfactory	0.531	0.194-1.453		0.290	0.061-1.383	
Very satisfactory	Reference			Reference		
**Access to Research-Related Institutional Training and Workshops**						
Not at all	2.250	0.805-6.286	0.254	4.499	1.074-18.845	0.116
Somewhat	1.446	0.435-4.803		2.364	0.507-11.013	
Enough	Reference			Reference		

### 3.6 Regional distribution of suicidal ideation

**Fig 1:**
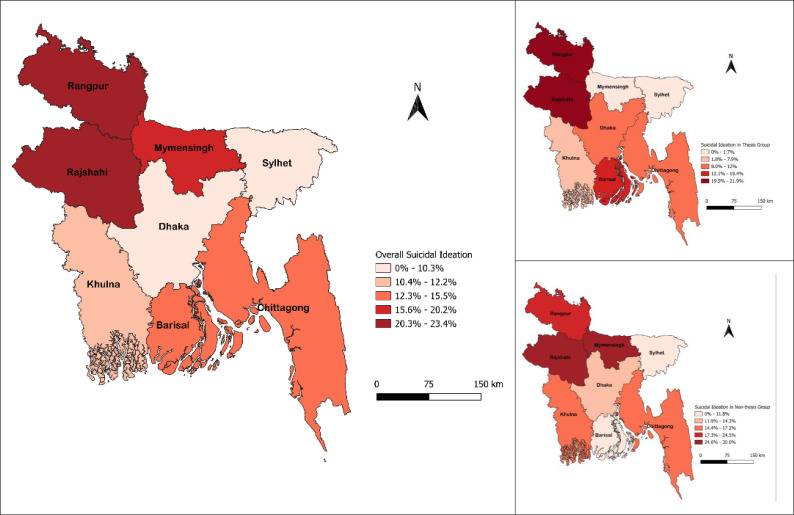
Geospatial distribution of suicidal ideation across administrative divisions of Bangladesh. The map was created by the authors using administrative boundary data from SimpleMaps (https://simplemaps.com/gis/country/bd), licensed under the Creative Commons Attribution 4.0 International (CC BY 4.0). Visualization was produced using QGIS Desktop version 3.32.3.

Fig 1 represented the geographical distribution that visualizes the divisional distribution of suicidal ideation in Bangladesh. Although no significant association was observed in the overall sample between suicidal ideation and the divisions (χ² = 10.631, *p* = 0.156), some regions showed higher rates compared to others. The highest rates of prevalence of suicidal ideation were found among participants from Rajshahi (23.4%) and Rangpur (22.0%) followed by Mymensingh (17.6%), Barishal (15.0%), Chattogram (12.7%), Khulna (10.4%), Dhaka (10.2%), and Sylhet (0.0%). Post-hoc analysis based on respondent’s thesis status also revealed no statistical difference in the geospatial distribution of suicidal status among thesis (χ² = 9.101, *p* = 0.245) or non-thesis (χ² = 5.762, *p* = 0.568) participants.

## 4. Discussion

This study investigated the prevalence and associated factors of past-year suicidal ideation among Bangladeshi university students and graduates, with a particular focus on mental health conditions and research-related academic experiences. The findings revealed that 14.0% of the participants reported experiencing suicidal ideation in the past year, aligning with global estimates among university populations in low- and middle-income countries. Consistent with existing literature, anxiety and insomnia showed significant association with suicidal ideation, even after controlling for socio-demographic and academic variables. Notably, students engaged in thesis work had significantly lower odds of suicidal ideation, indicating an association between thesis engagement and lower likelihood of suicidal ideation. Given the cross-sectional nature of the study, this association should not be interpreted causally and may reflect selection processes or contextual academic factors rather than a direct effect of thesis engagement. Conversely, poor supervisor availability, lack of publication interest, and limited participation in research training were linked to elevated risk, indicating the role of academic mentorship and institutional research culture in shaping student mental health.

The prevalence of past-year suicidal ideation in this study was found to be 14.0%, which aligns closely with previous research conducted in Bangladesh. For example, a study at Bangabandhu Sheikh Mujibur Rahman Science and Technology University reported a prevalence of 14.7% [16], while similar rates were observed among students at Jahangirnagar University (13.8%) [17] and Dhaka University (13.4%) [15]. From a global perspective, the findings of this study concur with several studies including a meta-analysis which reported that 15.9% of university students from 12 Muslim majority countries had past year suicidal ideation [39]. Another meta-analysis by Wang et al. found a prevalence of 9.33% among Chinese university students [40], while a meta-analysis of college students with a median age of 21.4 years reported a 10.6% prevalence of past-year suicidal ideation [4]. Several cross-sectional studies also reflect comparable rates, for instance, 10% among students in Spain [41] and 15.2% in Ghana [42]. However, variations were observed, with some studies reporting lower prevalence rates and some other higher prevalence rates compared to this study. For example, lower prevalence was reported among Norwegian university students (7.2%) [43], whereas higher rates were found in other settings, such as 34% at Mettu University in Ethiopia [44], 42.2% among university students in the UK [45], and 40% among doctoral students in the UK as reported by Hazell et al. [9]. These global discrepancies likely reflect differences in academic stress, cultural norms, mental health stigma, and institutional support. Nonetheless, the prevalence identified in this study is broadly consistent with national findings, suggesting that the unique academic and psychosocial pressures faced by Bangladeshi students are key contributors to suicidal ideation risk.

Globally and nationally, sociodemographic factors such as gender [23,44,46], socioeconomic status [17], marital status [23], and permanent residence [44] have often been found to be significantly associated with mental health outcomes, particularly suicidal behaviors. However, in the present study, none of these variables showed a significant association with suicidal ideation. This finding is consistent with several previous studies in the Bangladeshi context, where gender [15,16,22,47], permanent residence [16,17,22,23], socioeconomic status [16,23], age [48], and marital status [49] were also not significantly associated with suicidal ideation among university students. One possible explanation for the lack of association in the current study may be the shared campus environment and close peer interactions among university students, which could foster opportunities for social support and open communication. Such shared environments may attenuate the influence of individual socio-demographic differences on suicidal ideation risk. This interpretation is supported by findings from Miranda‐Mendizabal et al. [41], who reported that support from family members and peers played an important role, particularly among female students, in reducing the likelihood of suicidal ideation.

Students often face considerable pressure during their thesis work, as it is perceived to be a critical indicator of academic achievement and a gateway to future career opportunities. Factors such as tight deadlines, high expectations from supervisors, fear of failure, and academic stress have been shown to contribute to feelings of hopelessness, which is a known mediator between self-esteem, social anxiety, and suicidal ideation [50]. For instance, Zhang documented 61 suicides among doctoral students in China between 2010 and 2022, attributing them to factors including supervisor pressure, delayed graduation, mental and physical abuse, financial difficulties, and even sexual harassment [28]. Similarly, another study found that abusive supervision contributed directly to suicidal ideation and indirectly through perceptions of thwarted belongingness and burdensomeness [29]. Several global studies have reported a high prevalence of suicidal ideation among students in intensive research programs, including postgraduate researchers in the UK [8], doctoral students in UK [9], and PhD students of Economics in USA [7]. In addition, Hazell et al. reported that 40.3% of PhD students experienced mental health problems, with 42.3% viewing such problems as “the norm” and 35.8% having considered ending or pausing their program [51]. A multi-country study involving PhD students from 54 countries found that satisfaction with research programs was moderately and negatively correlated with depression and stress, particularly during the dissertation phase [52].

In contrast, the present study found that thesis engagement among Bangladeshi university students and recent graduates was associated with significantly lower odds of suicidal ideation. This pattern may reflect differences in the structure of thesis work and the criteria for student selection within the Bangladeshi higher education system. Thesis projects in this context are typically short-term and less intensive than multi-year doctoral programs and are generally available only to high-performing students. Most departments require a minimum CGPA of 3.00–3.50 for thesis eligibility, and none allow access to students with a CGPA below 3.00. Additionally, the only national fellowship program, the NST Fellowship, which supports master’s-level theses, enforces strict eligibility criteria, including a CGPA of 3.40 and restricting funds for science and technology related disciplines [53]. Due to these academic requirements, many students are unable to enroll in thesis-based master’s programs or secure thesis opportunities. This exclusion may undermine students’ confidence, limit career and higher education prospects, reduce access to faculty mentorship, and foster feelings of inadequacy, all of which may contribute to higher suicidal ideation among non-thesis students. For instance, students with lower CGPAs often feel helpless and concerned about employment prospects [30], and they frequently encounter difficulties securing funding for overseas study [54]. Conversely, thesis students may benefit from structured academic routines, enhanced supervision, peer collaboration, and a stronger sense of purpose, all of which may be associated with better mental health. Given the cross-sectional design, these associations should be interpreted cautiously and not as evidence of a protective or causal effect of thesis engagement. These findings highlight the need to expand equitable research opportunities and ensure that academic exclusion does not exacerbate mental health risks.

Many participants in the current study reported challenges such as limited supervisor availability, unclear academic guidance, and inadequate support in preparing for publication, which were significantly associated with higher levels of suicidal ideation in the unadjusted analysis. These findings align with global literature emphasizing the harmful effects of poor supervisory relationships on student well-being. For example, one study found that strained supervisor interactions were significantly associated with depression and suicidality among postgraduate researchers in the UK [8], while another reported that abusive supervision contributed both directly and indirectly to suicidal ideation among graduate students in China [29]. Another study also documented that the relationship between students and supervisors is a significant predictor of the psychological wellbeing of doctoral students in the UK [55]. Notably, participants who were not interested in publishing their thesis work had significantly higher odds of suicidal ideation compared to those expressing publication interest. This reluctance may reflect academic disengagement or reduced research self-efficacy, particularly in contexts where students face structural challenges such as inadequate funding, limited institutional support, and insufficient supervisory responsiveness. Consistent with this pattern, dissatisfaction with supervisor availability and responsiveness was also associated with a markedly elevated risk of suicidal ideation. Although a majority of participants perceived some level of institutional support, only a small fraction described their research environment as highly supportive, indicating a gap between basic access and meaningful academic mentorship. Prior studies have shown that insufficient faculty engagement, limited access to research training, and a lack of structured feedback can foster academic isolation and psychological distress among students [28,51]. In academic systems where research participation is competitive and mentorship structures are inconsistently applied, these institutional and supervisory gaps may exacerbate feelings of inadequacy and helplessness. However, some adjusted estimates showed wide confidence intervals, indicating imprecision, and these associations should therefore be interpreted cautiously. These findings highlight the urgent need for higher education institutions to cultivate a research culture grounded in accessible mentorship, clear academic guidance, and equitable support systems to help prevent suicidality among student researchers.

In the adjusted model, depression was not significantly associated with suicidal ideation, although it showed a significant relationship in the unadjusted analysis (OR = 2.51). This pattern is consistent with findings by Mamun et al. [16], where depression predicted suicidal ideation before adjusting for confounders. Such differences are expected, as adjusted models account for the influence of multiple variables. This attenuation may reflect shared variance between depression, anxiety, and insomnia, which are closely related symptom domains, with anxiety and sleep disturbances showing more proximal associations with suicidal ideation in the adjusted model. Similarly, Wang et al. [40] observed a reduced association between depression and suicidal ideation when controlling for additional factors. However, other studies reported a persistent association; for example, a study reported a moderate to strong positive correlation between depression and suicidal behaviors among postgraduate researchers in the UK [8], which was also confirmed in a larger study using the same population [56]. These discrepancies across studies may be attributable to differences in sample composition, severity of depressive symptoms, academic context, and analytic approaches. In contrast, anxiety remained a significant predictor in the present study, with anxious participants being 2.41 times more likely to experience suicidal ideation. This finding aligns with a systematic review and meta-analysis from Brazil [57], a study among university students in southern Bangladesh [23], cross-national research involving Chinese and U.S. college students [58], and post graduate researchers in UK [8]. Additionally, insomnia emerged as a significant risk factor, with affected students being 3.3 times more likely to report suicidal ideation. This result is consistent with Brailovskaia et al. [59], who found a significant link between insomnia and suicidal ideation among university students, and with a meta-analysis reporting an elevated risk by 2 times among insomniac students [60]. Taken together, these findings highlight the complex interplay between affective symptoms and sleep disturbances in shaping suicidal ideation, indicating the need for targeted mental health screening and support interventions in university settings.

The geospatial analysis revealed no statistically significant differences in the prevalence of suicidal ideation across the eight divisions of Bangladesh. However, as an exploratory component, spatial visualization can still provide contextual insights, highlight potential spatial clustering, and support hypothesis generation for future studies. It provides an integrated descriptive approach by enabling surveillance at the local or regional level and may help inform health policy discussions, particularly regarding regional mental health priorities [61]. In the present study, comparatively higher rates were observed in the northern and north-western regions, particularly in Rangpur, Mymensingh, and Rajshahi. This pattern aligns with a previous study among students taking university entrance tests in Bangladesh, which reported elevated suicide rates in both the northern and capital regions [62]. The observed regional disparities may be partially explained by underlying socio-economic inequalities, as a substantial portion of the population in Rangpur, Rajshahi, and Mymensingh divisions live below the poverty line [63]. These contextual vulnerabilities may contribute to increase psychological distress, although no causal inferences can be drawn from the current analysis.

The findings of this study highlight several important areas for intervention to address suicidal ideation among university students and graduates in Bangladesh. Mental health conditions, particularly anxiety, and insomnia showed significant association with suicidal ideation, while depression demonstrated a strong unadjusted relationship, underscoring the importance of strengthening mental health services within academic institutions. Universities may consider prioritizing the development of accessible counseling services and tailored mental health programs. Specific interventions could include stress management workshops, sleep hygiene education, and peer-led support groups to reduce stigma and foster a more supportive academic environment. Furthermore, the study emphasizes the importance of equitable academic opportunities, particularly for non-thesis students who were found to be to have higher odds of suicidal ideation. Institutions may consider reviewing restrictive policies that limit thesis access based solely on academic performance, with the aim of promoting broader student engagement in research. Providing structured mentorship, inclusive research environments, and career development support may contribute to reducing feelings of inadequacy and enhance student well-being. Additionally, the study identified key institutional and supervisory factors associated with suicidal ideation. Students who reported poor supervisor availability, unclear guidance, or lack of support for publication had significantly higher prevalence of suicidal ideation. These findings suggest the need for improved supervisory training and structured support systems to foster healthier faculty-student relationships. Moreover, although exploratory and not statistically significant, geospatial patterns suggesting relatively higher prevalence of suicidal ideation in the northern divisions of Bangladesh highlight the potential value of region-specific mental health initiatives to address contextual disparities in support access and socio-economic stressors.

Despite its strengths, this study has several limitations. Its cross-sectional design limits causal inferences, and the use of non-probability sampling may introduce selection bias and limit representativeness, affecting the generalizability of the findings. Accordingly, the results should be interpreted as sample-specific rather than nationally representative of Bangladeshi university students and graduates. Additionally, reliance on self-reported data could result in recall bias and under- or over-reporting of sensitive experiences. Moreover, suicidal ideation was assessed using a single-item binary measure, which does not capture frequency, severity, duration, or planning; therefore, it may underestimate the complexity of suicidality and limit comparability with studies using multi-item validated scales. Some regression estimates also showed wide confidence intervals, indicating imprecision; given conceptual overlap among depression, anxiety, and insomnia, adjusted associations should be interpreted cautiously, focusing on direction and magnitude rather than statistical significance alone. In addition, the geospatial analysis was descriptive and exploratory, and no statistically significant divisional differences were observed; therefore, the mapped patterns should not be interpreted as inferential evidence of regional disparities. Despite these limitations, the study provides novel insights into research-related academic stressors and suicidal ideation among an underexplored population.

Future research should incorporate longitudinal designs to explore causal pathways between academic stress, mental health challenges, and suicidal ideation. Using validated multi-item measures of suicidal ideation and related constructs could enhance the robustness of the findings in future studies. To reduce inherent sampling bias, ensure representativeness, and enhance generalizability of the findings, future research should incorporate a probability sampling technique, such as simple random sampling, stratified random sampling, or cluster random sampling. Qualitative studies are also recommended to gain deeper insights into the lived experiences of students—particularly those excluded from thesis tracks—and to identify specific academic stressors contributing to psychological distress. Expanding research across diverse educational and cultural settings would further enhance generalizability and support the development of context-specific interventions to reduce suicide risk among students globally. Given the conceptual overlap among mental health symptoms, future studies should continue to interpret multivariable findings with appropriate caution.

## 5. Conclusions

This study highlights the considerable burden of suicidal ideation among university students and recent graduates in Bangladesh, identifying associated academic, psychological, and institutional risk factors. Mental health conditions, particularly anxiety and insomnia showed significant associations, while academic factors, particularly non-thesis status, poor supervisory support, and less supportive research environments, also emerged as important correlates of suicidal ideation. The higher odds observed among non-thesis students suggests that limited access to research engagement may be associated with greater psychological vulnerability, although no causal inference can be drawn from this cross-sectional design. Perceived gaps in mentorship and institutional support further indicate the need for a more supportive academic structure. To address these challenges, universities may consider adopting inclusive and equitable research policies, review restrictive thesis eligibility criteria, expand mental health services, and foster a culture of empathetic, structured supervision. Moreover, universities may consider strengthening logistical assistance, career development support, and research training opportunities to promote a more supportive academic environment. Future studies using longitudinal and qualitative designs would be valuable to better understand how academic pressures and research-related experiences shape mental health over time. Evidence-based interventions tailored to the academic context may play an essential role in reducing suicidality and promoting student well-being, especially in resource-limited settings such as Bangladesh.

## Supporting information

S1 Data Dataset. (SAV)
